# Gene Therapy for Inherited Hearing Loss: Updates and Remaining Challenges

**DOI:** 10.3390/audiolres13060083

**Published:** 2023-12-04

**Authors:** Roni Hahn, Karen B. Avraham

**Affiliations:** Department of Human Molecular Genetics and Biochemistry, Faculty of Medicine and Sagol School of Neuroscience, Tel Aviv University, Tel Aviv 6997801, Israel; hahnroni@mail.tau.ac.il

**Keywords:** genetics, genomics, deafness, inner ear, cochlea, delivery, editing

## Abstract

Hearing loss stands as the most prevalent sensory deficit among humans, posing a significant global health challenge. Projections indicate that by 2050, approximately 10% of the world’s population will grapple with disabling hearing impairment. While approximately half of congenital hearing loss cases have a genetic etiology, traditional interventions such as hearing aids and cochlear implants do not completely restore normal hearing. The absence of biological treatment has prompted significant efforts in recent years, with a strong focus on gene therapy to address hereditary hearing loss. Although several studies have exhibited promising recovery from common forms of genetic deafness in mouse models, existing challenges must be overcome to make gene therapy applicable in the near future. Herein, we summarize the primary gene therapy strategies employed over past years, provide an overview of the recent achievements in preclinical studies for genetic hearing loss, and outline the current key obstacles to cochlear gene therapy.

## 1. Introduction

Hearing loss affects hundreds of millions of people around the world as the most common human sensory disorder. While deafness already presents a significant global public health concern, with approximately 1 in 500 newborns, its prevalence is projected to escalate markedly [1]. By 2050, the number of individuals who experience disabling hearing impairment is expected to double and surpass 900 million people [2]. About 50% of congenital hearing loss cases can be attributed to genetic causes. These genetic factors can lead to the condition manifesting either at birth or later in life, exhibiting a range of severity levels [3,4]. In recent decades, significant progress in understanding the genetic basis of deafness has been made, leading to the discovery of more than 150 genes [5]. Despite these significant advancements, the field still lacks clinical interventions that can fully restore natural hearing. Current approaches, like hearing aids and cochlear implants, successfully enhance auditory abilities within specific patient populations, but they fall short of providing therapy to reinstate the inner ear’s original functionality completely [5,6,7].

**Figure 1:**
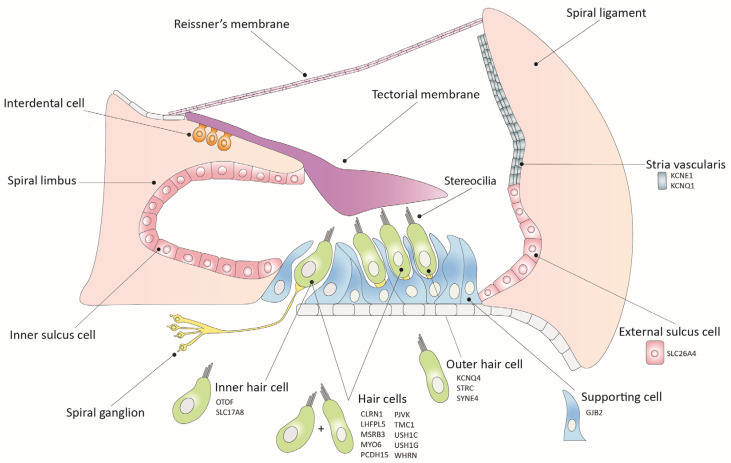
Schematic illustration of the organ of Corti, including the expression sites of causal genes of hearing loss. All genes are part of proof-of-concept studies for inner ear gene therapy.

The imperative need to find biological treatments for genetic deafness has spurred a global undertaking over the past two decades. This effort has led to the development of preclinical therapies for genetic hearing loss, with over 40 reported studies, including more than 20 deafness genes [8]. These studies have demonstrated success in restoring hearing loss attributed to various mutations and genes across diverse stages of development (Figure 1). While the potential application of these therapeutic strategies to humans is now within closer reach than ever, substantial challenges remain on the horizon. These include optimizing the treatment’s transduction efficiency and specificity through more targeted capsids and promoters, identifying superior delivery methods, and a deeper comprehension of potential treatment side effects [9,10].

Moreover, a notable obstacle lies in the genetic diversity inherent in deafness. Each gene linked to deafness, along with its unique pathogenic variations, represents only a segment within the intricate tapestry of genetic hearing impairments [11]. This diversity necessitates a diverse array of gene-specific therapies and complicates the implementation of treatments tailored to specific variants.

In this review, we provide an overview of the significant advances in gene therapy for hearing loss, shedding light on the prevailing obstacles that require resolution from the scientific community.

## 2. Inner Ear Gene Therapy Strategies

Genetic therapy is a research field that aims to modify, replace, or repair the genetic factors responsible for genetic abnormalities. Advancements in gene delivery technologies and therapeutic tools, as well as increasing knowledge about the genes involved in hearing loss and their functions in the auditory system, have led to a rapid expansion in the field of gene therapy for heredity hearing loss [12]. The inner ear is particularly well-suited for such interventions due to its unique properties. Firstly, the ear comprises distinct compartments that allow for the targeted delivery of therapeutic agents with minimal diffusion beyond the surrounding tissues. Secondly, the ear contains fluids that facilitate the dissemination of therapeutic agents to many target cells if administered locally. Various types of genetic therapies, including gene replacement, genome editing, and RNA-based therapies, can be used for this purpose [6,13,14].

### 2.1. Gene Replacement

The gene replacement approach, the most common strategy in gene therapy experiments in the inner ear, involves delivering a functional coding sequence of cDNA for a specific gene into targeted cells to replace a nonfunctional mutant gene and restore its normal function. In cases of recessive inheritance, where two copies of the mutated gene are present, gene replacement can supplement the nonfunctional mutant gene with a functional copy. In dominant inheritance with haploinsufficiency, where one copy of the gene is insufficient for normal function, gene replacement can provide an additional functional copy of the gene [15,16]. Delivery timing plays a critical role in the effectiveness of gene replacement. If the expression of the transgene begins during the prenatal stage, the lack of a functional gene can result in significant developmental consequences that cannot be reversed through introducing the functional sequence at later stages. Although many successful studies have been shown in different animal models of genetic deafness for various stages of inner ear maturation, most of these studies have been conducted during early neonatal stages (Table 1) [17].

### 2.2. Gene Suppression

Gene suppression therapies involve the use of small RNA molecules, such as small interfering (siRNAs), microRNAs (miRNAs), and antisense oligonucleotides (ASOs), to specifically silence genes without affecting the DNA sequence. While ASOs are designed to target a single mRNA molecule and bind to the RNA directly, siRNAs or miRNAs trigger cellular pathways that lead to the degradation of the RNA target. RNA gene suppression therapies are particularly relevant for dominant mutations in heterozygous models (Table 2). In such cases, silencing the mutated gene while retaining the expression of the wild-type gene may be adequate to counteract the effects of the dominant form of hearing loss [47,48,49].

Gene suppression involving the use of RNAi (RNA interference) was performed in Beethoven (*Bth*) mice, a mouse model of TMC1 (transmembrane channel-like 1) autosomal dominant hearing loss [50] (Table 2). An AAV vector containing a synthetic microRNA specifically designed to target the dominant allele was inserted through a single intracochlear injection. Some of the treated mice exhibited slower deterioration of hearing, with ABR thresholds similar to wild-type mice [51]. In addition to conventional RNA interference techniques, a novel and highly specific RNA interference tool, the CRISPR-Cas13 RNA editing system, has been used recently in neonatal *Bth* mice. This proof-of-concept study revealed a remarkable 70% reduction in *Tmc1 Bth* mRNA in vivo using the CRISPR-CasRx system, accompanied by minimal off-target effects [52].

One example of the therapeutic potential of ASOs was shown in a mouse model of Usher Syndrome type 1 [53,54,55]. The systemic administration of antisense oligonucleotides was investigated in a mouse model carrying a c.216G>A variant of the *Ush1c* gene, with three localized delivery strategies. The study reported a significant improvement in auditory and vestibular function with ASO through inner or middle ear injection [56].

These observations, along with the approval of other nucleic acid drugs and ongoing clinical trials as potential therapeutics for various diseases, have generated significant interest in developing oligonucleotides and nucleic acids for genetic hearing loss [57].

**Table 2:** Summary of gene suppression preclinical therapy studies, using mice models.

Gene (Deafness Form)	Animal Model	Hearing Impairment	Strategy	Injection Age	Injection Route	Vector	Reference
*GJB2* (DFNA3A)	*Gjb2* p.R75W	Severe to profound	RNAi	P42–P45	RWM	siRNAs	[49]
*TMC1* (DFNA36)	*Tmc1-Bth*	Progressive	miRNA	P0–P2	RWM	AAV9	[50]
	*Tmc1-Bth*	Progressive	miRNA	P15–P16; P56–P60; P84–P90	RWM, PSCC	AAV9	[51]
	*Tmc1-Bth*	Progressive	RNA editing (CasRx)	P1–P2	RWM	AAV9-PHP.eB	[52]
*USH1C* (USH1C)	*Ush1c* c.216G>A	Severe (balance defect)	Antisense oligonucleotide	P1; P3; P5; P7	Intraperitoneal	ASO-29	[58]
	*Ush1c* c.216G>A	Severe (balance defect)	Antisense oligonucleotide	P1; P5; P10; P20	RWM	ASO-29	[56]
	*Ush1c* c.216G>A	Severe (balance defect)	Antisense oligonucleotide	E12.5	EUGO	ASO-29	[59]

EUGO—electroporation-mediated transuterine gene transfer into otocysts; RWM—round window membrane; P—post-natal day; PSCC—posterior semicircular canal.

### 2.3. Gene Editing

Gene editing techniques enable targeted modifications to the genome, including the deletion, addition, and replacement of bases to repair mutations and restore the wild-type function. Previously, gene modification methods relied mainly on technologies such as zinc-finger nucleases and TALENs that utilized specific protein motifs to bind to a particular genomic DNA sequence, inducing double-strand break (DSB) and repair mechanisms. However, a gradual advancement in genome editing tools has resulted in higher performance levels. Currently, two primary gene editing technologies, CRISPR-Cas and base editing, are widely used for inner ear applications [60,61] (Table 3). The clustered regularly interspaced short palindromic repeat (CRISPR) system involves using a small piece of RNA to guide a nuclease enzyme, Cas9, to a specific location in the genome where it cuts the double-stranded DNA. The cut triggers the cell’s natural DNA repair mechanisms, which can be harnessed to either insert, delete, or replace DNA at the cut site. Due to its precise performance, easy use, and low price, the CRISPR-Cas system has significantly replaced earlier methods [62,63].

Gene editing using the CRISPR-Cas method has been applied in a mouse model of human DFNB23 deafness, serving as an example of its practical application (Table 3). This model has a frameshift mutation in the *Pcdh15* transcript due to the insertion of a single base. Through injecting a gRNA that mainly causes a 1-bp deletion, the frameshift mutation in the hair cells of the mutant mice was corrected. As a result, the auditory and balance functions of the mice were partially rescued [64].

Another approach to performing gene editing is base editing, a technique for creating targeted single-nucleotide alterations without generating a double-strand break (DSB) of the DNA. Base editing uses a modified version of Cas9, and a second enzyme, such as a cytidine or adenine deaminase, to directly convert one DNA base to another [65,66]. One example of base editing with direct repair was conducted on *Tmc1* mice, aimed to correct the Baringo mutation to the wild-type sequence using a cytosine base editor. A dual-AAV system was used to deliver the editor to prevent hearing loss. Successfully, the intervention managed to reverse 51% of the point mutation and led to a notable improvement in hearing function [67]. RNA base editors can offer a correction on the RNA level and, therefore, present an elevated safety profile for the remediation of genetic disorders.

A study using RNA-based editors to treat hereditary hearing loss was performed in *Myo6* mice carrying a dominant mutation (Table 3). To correct the mutated allele, a mini dCas13X.1 base editor was used, effectively restoring the mice’ auditory function for up to three months [68]. Gene correction indeed holds substantial promise in the realm of inner ear therapy. However, several challenges persist that require resolution, notably on the optimization of editing efficiency, as well as reducing off-target effects.

**Table 3:** Summary of gene editing preclinical therapy studies, using mouse models.

Gene (Deafness Form)	Animal Model	Hearing Impairment	Strategy	Injection Age	Injection Route	Vector	Reference
*KCNQ4* (DFNA2A)	*Kcnq4* c.827G>C	Progressive	Disruption	P0–P1	PSCC, RWM, utricle, scala media	Dual vector: Anc80L65	[69]
	*Kcnq4* c.683G>A	Progressive	Disruption	P1–P2	Scala media	AAV9-PHP.eB	[65]
MYO6 (DFNA22)	*Myo6* p.C442Y	Progressive	Disruption	P0–P2	Scala media	AAV9-PHP.eB-	[70]
	*Myo6* p.C442Y	Progressive	RNA base editing	P0–P2	Scala media	AAV9-PHP.eB (RNA ABE)	[68]
PCDH15 (DFNB23/USH1F)	*Pcdh15*av−3J	Profound (balance defect)	Frame restoration	P0–P2	Scala media	AAV9	[64]
TMC1 (DFNA36)	*Tmc1-Bth*	Progressive	Disruption	P1	Utricle	Dual vector: AAV9-PHP.B	[40]
	*Tmc1-Bth*	Progressive	Disruption	P1–P2	Inner ear	Dual vector: Anc80 L65	[71]
TMC1 (DFNB7/11)	*Tmc1*-Baringo	Profound	Base editing	P1	Inner ear	Dual vector: Anc80 L65 (CBE)	[67]

P—post-natal day; PSCC—posterior semicircular canal; RWM—round window membrane.

## 3. Delivery Vectors

### 3.1. Adeno-Associated Virus

After more than 50 years of research, the adeno-associated virus (AAV) has gained recognition as a highly effective and versatile human gene transfer tool. With more than 170 clinical trials, AAV has demonstrated its tremendous potential and has been utilized as a gene therapeutic, as seen in Luxturna, Glybera, and Zolgensma [72,73,74]. AAV, a member of the *Parvoviridae* family, is a small, non-pathogenic virus with a single-stranded DNA genome. AAV can be engineered to target specific cell types and has high transduction efficiency and long-term gene expression in both animal models and humans [75,76]. The AAV capsid exhibits an icosahedral symmetry with a diameter ranging from 18 to 28 nanometers. It is responsible for enclosing a single-stranded (ss) DNA genome that is approximately 4.7 kilobases in size. The DNA is flanked by two inverted terminal repeats (ITRs), crucial for viral genome replication, encapsidation, and integration into the host cell genome [77]. During recombinant AAV (rAAV) production, the therapeutic gene expression cassettes replace the wild-type AAV open-reading frames, preserving the essential inverted terminal repeat sequence (ITR) viral sequences. The elimination of viral coding sequences enables the minimization of the immunogenicity and cytotoxicity of the vector when introduced in vivo [78,79]. AAV’s different serotypes are another critical feature that contributes to their versatility. Researchers have identified thirteen naturally occurring AAV serotypes and over 100 additional variants from various animal species. Each serotype has distinct capsid structures that affect its ability to transduce specific cell types and tissues [80]. AAV vectors exhibit considerable potential as a gene therapy tool for different diseases, and currently, they are the safest and most promising viral vectors for inner ear gene therapy.

To date, the effectiveness of gene delivery to cochlear hair cells has been notably observed with AAV vectors, specifically AAV2 and AAV9, as well as the synthetic constructs Anc80L65 and AAV9-PHP.eB. These vectors have demonstrated exceptional transduction efficiency and have consequently garnered favor among numerous research groups.

A significant limitation of AAV lies in its cargo capacity (4.7 kb), limiting the size of the therapeutic sequence [81,82,83]. Given that several deafness-related genes extend considerably in length, addressing this has led to the concept of co-transduction with two AAVs [29,38,40,84,85] or even a triple-AAV strategy [86]. A recent study in a mouse model of *PCDH15*-related hearing loss highlights an alternative strategy aimed at overcoming the restricted cargo capacity of AAV. Given that the full-length *Pcdh15* transcript spans 7.9 kb, surpassing the maximum delivery capability of AAV, single-AAV gene replacement becomes unfeasible. To address this, the concept of “mini-PCDH15” proteins was devised, involving the removal of extracellular domains under the assumption that specific domains might be redundant or dispensable for achieving partial hearing restoration. The results included preventing the degeneration of hair cell bundles and partial restoration of auditory brainstem response (ABR), emphasizing that complete protein expression might not be essential [31]. Expanding our knowledge of hair cells’ molecular mechanism and dynamics could pave the way for refining mini-gene replacement strategies for treating hereditary hearing loss.

### 3.2. Lentivirus

Lentiviruses (LVs), belonging to the *Retroviridae* family, are RNA viruses that use reverse transcriptase to convert their RNA genome into DNA. It comprises enveloped virions that contain a single-stranded DNA genome of approximately 9 kb. In addition to the large capacity size, LVs are considered a viable option for gene delivery applications as they can naturally infect various types of cells, including both dividing and non-dividing cells such as neurons and certain types of immune cells [87,88]. However, despite the benefits of using LVs, the risk of insertional mutagenesis, which can potentially disrupt gene function in transduced cells, is one of the main barriers to using lentiviral vectors for in vivo gene therapy [76,89]. Furthermore, LVs have not been extensively used in inner ear applications because they cannot specifically target hair cells [90,91]. However, generating viable lentiviral vector candidates for effective gene delivery in the inner ear may be possible with further advancements and modifications.

### 3.3. Adenovirus

Adenoviruses (AdVs) are a family of non-enveloped, double-stranded DNA viruses that can infect a wide range of animals, including humans. A major advantage of AdV is its large packaging capacity, with a range of 26 kb to 45 kb, depending on the serotype [92,93]. However, the use of adenovirus vectors derived from the most prevalent serotypes is limited due to their prevalence among healthy individuals. As a result, researchers have instead turned to using rare serotypes such as 2 and 5 to develop AdV vectors for gene therapy. Still, the highly immunogenic nature of all AdV proteins raises additional concerns [94,95]. A further limitation of AdVs is the lack of integration into the host genome, resulting in a short duration of transgene expression that typically lasts only weeks to months [96]. This evidence makes it less suitable for rescuing genetic hearing loss, which typically requires long-term transgene expression, but it is more convenient for regeneration and otoprotection applications.

## 4. Inner Ear Delivery Approaches

Inner ear gene therapy routes are integral to the development of innovative treatments for hearing and balance-related disorders. Each route has its own advantages and considerations, while choosing the most suitable path depends on factors such as the nature of the disorder and the desired treatment outcome.

### 4.1. Round-Window Injection

**Figure 2:**
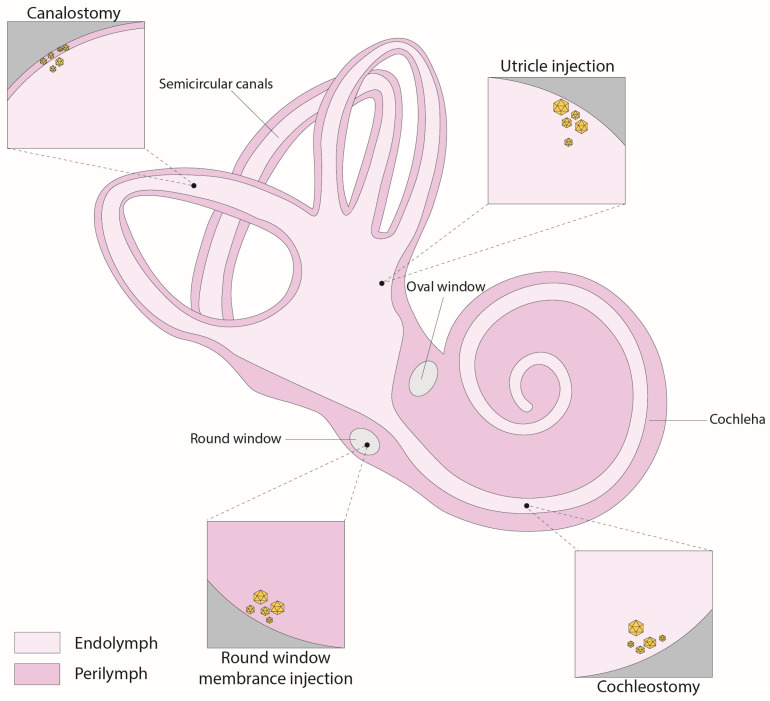
Schematic illustration of inner-ear gene therapy routes.

The round-window membrane (RWM) is a three-layered flexible membrane that opens to the perilymph space of the scala tympani. This intracochlear method has become the most commonly used method for delivering genetic agents into the inner ear (Figure 2). Compared to other methods, the RWM approach is considered relatively safe and minimally invasive, with a low risk of hearing damage, as only the membrane needs to be pierced, and the perilymph volume is larger than the endolymph [97]. Additionally, studies have demonstrated the successful transduction of both inner and outer hair cells, supporting cells, and spiral ganglion cells in non-human primates using AAV delivered via the round window [36,98]. One notable drawback of utilizing the RWM approach for gene therapy is the difficulty in achieving even distribution of the viral vector throughout the entire cochlear duct. Consequently, transduction often occurs in a gradient from the cochlear base to the apex, especially in adult mice. To address this challenge, a study utilized a combined approach of RWM injection along with canal fenestration to create an exit pathway [99]. This innovative strategy facilitated viral vectors to achieve comprehensive transduction of both cochlear inner hair cells (IHCs) and vestibular hair cells (VHCs), all the while avoiding any auditory dysfunction.

### 4.2. Canalostomy

An alternative method for delivering genetic agents into the inner ear is the canalostomy, mainly through the posterior semicircular canal (Figure 2). This technique involves creating an opening in the bony canal and introducing the therapeutic agent into the perilymphatic space. While this approach has proven successful in various preclinical studies [24,35,39,46], the challenge associated with this method is in achieving precise injections because of the small size of the semicircular canal, making it difficult to definitively ascertain whether the therapeutic product was delivered to the perilymphatic or endolymphatic compartments [100,101]. Adapting this method for use in humans could also prove challenging due to the anatomical positioning of the posterior semicircular canal. However, an alternative option could involve injection through the lateral semicircular canal in the human inner ear, provided that this approach has been previously validated as safe through experimentation in larger animal models [102,103].

### 4.3. Cochleostomy

The cochleostomy route delivers vectors directly into the scala media, a fluid-filled compartment within the cochlea (Figure 2). This is achieved through creating an opening through the basal part of the cochlea, allowing access to the cochlear endolymphatic space in close proximity to the round window. The safety and effectiveness of this approach have been demonstrated in neonatal mice and successfully applied in various preclinical studies [65,68,70,104,105,106]. Nevertheless, this surgical procedure is more intricate in adult mice because the cochlear bone is less pliable than in neonatal mice, resulting in a lasting elevation of hearing thresholds, particularly at high frequencies [105,107]. Consequently, this technique becomes invasive when applied to adult mice, potentially disrupting the cochlear structure or upsetting inner ear equilibrium through intermixing endolymph and perilymph fluids. Nonetheless, the larger size of the human cochlea could offer possibilities for future interventions in human patients, though additional validation is imperative.

### 4.4. Utricle

The utricle injection route was developed through the effort to target the endolymphatic fluids specifically (Figure 2). This approach efficiently delivered AAV vectors to all six sensory organs within the inner ear of neonatal mice. Administering injections through this route, readily accessible in neonatal mice, resulted in a remarkable rate of transduction IHCs and various other cell types within the inner ear, approaching nearly 100%. These injections did not cause any harm to auditory or vestibular functions [108]. Notably, when treatment was delivered via utricle injection to a mouse model with an *Strc* mutation, it led to a significant outcome; namely, partial prevention of hearing loss and restoration of stereocilia morphology in 50% of the treated animals [38]. Nonetheless, a limitation of this method lies in the challenge of definitively confirming whether the therapeutic products remain confined solely to the endolymphatic spaces within the inner ear or whether some reach the perilymphatic fluids. Moreover, it is important to highlight that the endolymphatic utricular area presents more complexities in humans, as it is primarily covered by the facial nerve, making access less straightforward [17].

## 5. Challenges and Limitations

### 5.1. The Mouse as a Model for Human Deafness

In inner ear research, mice models stand out as the prevailing choice for experimental animals due to their anatomical similarities to the human inner ears and extensively studied genomes [109]. Mice have played a pivotal role in unraveling numerous intricate processes and accumulating a vast wealth of knowledge about this organ, including examining different aspects of a gene therapy cure, such as delivery methods, the selectivity of vectors, and efficiency. Despite many preclinical trials that have successfully demonstrated the feasibility and potential of gene therapy as a treatment for hearing loss, one critical factor that needs to be considered is the dissimilar developmental timelines observed in mice and humans [110]. While mice are born with an underdeveloped cochlea that necessitates an additional two to three weeks to achieve full maturity, in humans, the auditory system is fully operational at birth, with auditory startle reflexes emerging at approximately 24 weeks of gestation. Consequently, most of the cochlear maturation in humans occurs in the prenatal stage [111,112]. Given that a significant portion of preclinical studies have been carried out in mice before the onset of hearing, the translation of these findings to a relevant therapeutic window in humans aligns with the gestational stage of around 20 weeks. Such intervention during pregnancy introduces a range of possible risk factors and ethical considerations, adding complexity to obtaining approval from regulatory bodies such as the FDA and EMA. Despite the promising results obtained from mouse studies for potentially treating genetic causes of deafness, the lack of clinical trials with positive outcomes presents a significant gap. Consequently, it remains a challenge to predict the potential consequences for humans at this juncture confidently.

### 5.2. Genetic Heterogenicity

One of the significant challenges in addressing genetic hearing loss arises from its inherent heterogeneity. More than 150 genes, including thousands of distinct variants, are known to be associated with hearing loss. Each of these genes presents unique complexities regarding protein function, the optimal timing for therapeutic intervention, and the specific target cell populations for treatment [3]. Genetic factors’ diversity necessitates a tailored and precise approach for each case. This complexity makes it challenging to devise a single, standardized gene therapy strategy that effectively addresses the broad spectrum of genetic causes of hearing loss. As medicine trends towards greater personalization, the effectiveness of precision medicine strategies in precisely targeting individual genetic variants remains uncertain.

### 5.3. Applications in Mature Mice

Most proof-of-concept studies aimed at restoring hearing loss have been conducted on neonatal mice during their early developmental stages. Performing interventions in adult mice is complicated due to two main factors. Firstly, the cochlea, which is the auditory portion of the inner ear, is embedded within the temporal bone, making it structurally difficult to access. Secondly, the effectiveness of gene delivery using viral vectors in the inner ear cells of mature mice is notably low, with a reduction in the effective transduction of OHC [113]. Nonetheless, a recent study may have provided a potential solution to tackle these obstacles. A recently explored novel approach enables drug delivery to the adult inner ear using cerebrospinal fluid (CSF) [34]. The brain and inner ear fluid are connected via the cochlear aqueduct, which allows the delivery of AAV-*Slc17a8* to adult *Slc17a8^−/−^* mice. Hearing recovery was observed two weeks post treatment, with sensory cells in the cochlea effectively transduced. This transduction was more pronounced at the cochlear base and gradually decreased towards the apex. While CSF delivery shows promise in facilitating treatment during later developmental stages, additional actions, such as using nonhuman primate models, are required.

Another critical aspect to consider when addressing hearing loss in adult mice is the variability of hearing upon aging [114]. This includes the presence of a variant allele in the *Cdh23* gene, which is associated with age-related hearing loss (ARHL) [115]. The effect of this variant on hearing loss has been evaluated in C57BL/6J and C57BL/6N strains [116]. This allele was corrected using CRISPR in a C57BL/6N strain, to alleviate this confounding problem of ARHL in preclinical studies [117]. The presence of this or other alleles leading to ARHL in mice should be considered when using a particular strain as a model for evaluation of hearing at post-natal stages.

### 5.4. Implications of the Immune Response

AAV-based approaches to restore auditory and vestibular function are the most promising and safest. Despite this advantage, our current understanding of the host immune responses to AAV within the mammalian inner ear remains limited [118,119]. Several critical questions must be addressed to bridge the gap between preclinical studies and clinical applications: are factors such as viral dose, serotype, and delivery route determining the degree of inflammation? Does pre-existing immunity influence the efficacy and safety of gene therapy within the inner ear? Answers to these questions will maximize the safety and effectiveness of human inner ear therapy. Insights from ongoing clinical trials centered on eye treatments have indicated that viral gene therapy might induce an adaptive immune response. This observation raises optimism for similar prospects in the inner ear domain [9,120,121].

## 6. Conclusions

Gene therapy holds great promise for restoring auditory function and treating hereditary hearing loss, a feat beyond the reach of conventional medical approaches. The collaborative efforts of numerous teams worldwide have resulted in several successful pre-clinical studies and, more recently, the initiation of phase 1/2 clinical trials for gene therapies targeting otoferlin-related hearing loss. These trials are being conducted by two companies, Akouos and Decibel Therapeutics [122,123]. Undoubtedly, the beginning of clinical trials is a considerable landmark in auditory research. However, it is essential to remain aware that numerous challenges must be addressed before genetic therapeutics can be applied to patients across the board with hereditary hearing loss. These challenges encompass addressing limitations such as transduction efficacy, assessing potential toxicity, and advancing the development of delivery methods that are both feasible and safe for human applications. In addition, a more comprehensive understanding of treatment side effects, including assessing the longevity of the treatment and potential immune responses, is imperative for the progress of gene therapy in this context. Together, the current advancements and forthcoming insights from clinical trials offer fresh optimism for addressing hereditary hearing loss.

## Figures and Tables

**Table 1:** Summary of gene replacement preclinical therapy studies, using mouse models.

Gene (Deafness Form)	Animal Model	Hearing Impairment	Injection Age	Injection Route	Vector	Pubmed
*CLRN1* (USH3A)	TgAC1/*Clrn1* KO	Delayed onset progressive	P1	RWM	AAV-S	[18]
	*Clrn1* KO	Profound	P1–P3	RWM	AAV2, AAV8	[19]
	*Clrn1e*^×4−/−^*/Clrn1* KO	Profound	P1–P3	RWM	AAV8	[20]
	*Clrn1* KO	Profound	P1	RWM	AAV9-PHP.B	[21]
*GJB2* (DFNA3A/DFNB1A)	*Gjb2* iCKO	Severe to profound	P28	RWM	Anc80L65	[22]
	*Gjb2* iCKO	Profound	P0, P42	RWM	AAV1	[23]
*KCNE1* (JLNS2)	*Kcne1* KO	Severe (balance defect)	P0–P2	PSCC	AAV1	[24]
*KCNQ1* (JLNS1)	*Kcnq1* KO	Severe (balance defect)	P0–P2	RWM, Scala media	AAV1	[25]
*LHFPL5* (DFNB66/67)	*Lhfpl5* KO	Profound (balance defect)	P1–P2	RWM, Scala media	exo-AAV1	[26]
*MSRB3* (DFNB74)	*Msrb3* KO	Profound	E12.5	EUGO	AAV1	[27]
*OTOF* (DFNB9)	*Otof* KO	Profound	P0–P2	RWM	Dual-vector: AAV9-PHP.eB	[28]
	*Otof* KO	Profound	P10, P17, P30	RWM	Dual vector: AAV2 quadY-F	[29]
	*Otof* KO	Profound	P6–P7	RWM	Dual vector: AAV6	[30]
*PCDH15* (DFNB23/USH1F)	*Pcdh15* KO	Profound (balance defect)	P1	RWM	AAV9-PHP.B	[31]
*PJVK* (DFNB59)	*Pjvk* G292R/G292R	Progressive (balance defect)	P0–P1	RWM	Anc80L65	[32]
	*Pjvk* KO	Variable (moderate to profound)	P3	RWM	AAV8	[33]
*SLC17A8* (DFNA25)	*VGlut3* KO	Profound	6–12 weeks	Cisterna magna	AAV9-PHP.B	[34]
	VGlut3 KO	Profound	5, 8, and 20 weeks	PSCC	AAV8	[35]
	VGlut3 KO	Profound	P1–P3 and P10–P12	RWM	AAV1	[36]
*SLC26A4* (DFNB4)	*Slc26a4* KO	Profound (balance defect)	E12.5	EUGO	AAV1	[37]
*STRC* (DFNB16)	*Strc* KO	Severe	P0–P1	Utricle	Dual vector: AAV9-PHP.B	[38]
*SYNE4* (DFNB76)	*Syne4* KO	Severe to profound progressive	P0–P1.5	PSCC	AAV2-9.PHP.B	[39]
*TMC1* (DFNB7/11)	*Tmc1* KO, *Tmc1* Baringo	Profound	P1, P7	Utricle	AAV9-PHP.B	[40]
	*Tmc1* KO, *Tmc1* N1931/N1931	Profound	P1	Utricle	AAV2-9-PHP.B	[41]
	*Tmc1* KO	Profound	P1–P2	RWM	Anc80L65	[42]
	*Tmc1* KO, *Tmc1 Bth*	Profound	P0–P2	RWM	AAV1	[43]
*USH1C* (DFNB18/USH1C)	*Ush1c* c.216G>A	Severe (balance defect)	P0–P1; P10–P12	RWM	Anc80L65	[44]
*USH1G* (USH1G)	*Ush1g* KO	Profound (balance defect)	P2.5	RWM	AAV8	[45]
*WHRN* (DFNB31)	*Whrn wi/wi*	Profound (balance defect)	P1–P5	PSCC	AAV8	[46]

EUGO—electroporation-mediated transuterine gene transfer into otocysts; KO—knock-out (gene-targeted mutagenesis); iCKO—inducible conditional Knock-out; P—post-natal day; PSCC—posterior semicircular canal; RWM—round window membrane.

## Data Availability

Not applicable.
